# Developing a platform for production of the oxylipin KODA in plants

**DOI:** 10.1093/jxb/erab557

**Published:** 2021-12-27

**Authors:** Yuta Ihara, Takayuki Wakamatsu, Mineyuki Yokoyama, Daisuke Maezawa, Hiroyuki Ohta, Mie Shimojima

**Affiliations:** 1 School of Life Science and Technology, Tokyo Institute of Technology, Yokohama 226-8501, Japan; 2 International Environmental and Agricultural Sciences, Tokyo University of Agriculture and Technology, Tokyo 183-8509, Japan; 3 Kishi Kasei Co. Ltd., Yokohama 236-0004, Japan; 4 University of Exeter, UK

**Keywords:** Allene oxide synthase, *Arabidopsis thaliana*, KODA, α-linolenic acid, 9-LOX, *Nicotiana benthamiana*, oxylipin

## Abstract

KODA (9-hydroxy-10-oxo-12(*Z*),15(*Z*)-octadecadienoic acid) is a plant oxylipin involved in recovery from stress. As an agrichemical, KODA helps maintain crop production under various environmental stresses. In plants, KODA is synthesized from α-linolenic acids via 9-lipoxygenase (9-LOX) and allene oxide synthase (AOS), although the amount is usually low, except in the free-floating aquatic plant *Lemna paucicostata*. To improve KODA biosynthetic yield in other plants such as *Nicotiana benthamiana* and *Arabidopsis thaliana*, we developed a system to overproduce KODA *in vivo* via ectopic expression of *L. paucicostata* 9-LOX and AOS. The transient expression in *N. benthamiana* showed that the expression of these two genes is sufficient to produce KODA in leaves. However, stable expression of 9-LOX and AOS (with consequent KODA production) in Arabidopsis plants succeeded only when the two proteins were targeted to plastids or the endoplasmic reticulum/lipid droplets. Although only small amounts of KODA could be detected in crude leaf extracts of transgenic *Nicotiana* or Arabidopsis plants, subsequent incubation of the extracts increased KODA abundance over time. Therefore, KODA production in transgenic plants stably expressing 9-LOX and AOS requires specific sub-cellular localization of these two enzymes and incubation of crude leaf extracts, which liberates α-linolenic acid via breakdown of endogenous lipids.

## Teaser

We established a system for KODA production in seed plants that takes advantage of the abundance of the substrate, α-linolenic acid, in vegetative tissues.

## Introduction

Fatty acids are essential constituents of glycerolipids such as phospholipids and galactolipids, which together comprise the majority of membrane lipids, and triacylglycerols, which are storage lipids (Mamode Cassim *et al.*, 2019; Wang *et al.*, 2019). Fatty acids newly synthesized in plastids are either saturated or monounsaturated, and their subsequent conversion to acyl-CoA enables the biosynthesis of phospholipids and galactolipids. The saturated/monounsaturated fatty acid esters in glycerolipids become desaturated to yield polyunsaturated fatty acid esters such as linolenoyl, linoleoyl, or roughanoyl esters (Li-Beisson *et al.*, 2013). Among the polyunsaturated fatty acids esterified to glycerolipids, α-linolenic acid is the most abundant in seed plants. This polyunsaturated fatty acid or its esters are useful compounds for biotechnological applications such as the production of docosahexaenoic acid and eicosapentaenoic acid (Han *et al.*, 2020).

Oxylipins are a large group of oxidized fatty acids, and their derivatives are mainly derived from polyunsaturated fatty acids such as linolenic acid, linoleic acid, and roughanic acid, or their esters in plants. Oxylipins include keto (oxo) fatty acids, epoxy fatty acids, hydroxy fatty acids, fatty acid hydroperoxides, divinyl ethers, aldehydes, and alcohols such as green leaf volatiles, jasmonic acid, and 12-oxo-phytodienoic acid (OPDA; Andreou *et al.*, 2009). Jasmonic acids are phytohormones that have diverse biological roles, such as in the response to wounding (Wasternack and Feussner, 2018). Moreover, certain green leaf volatiles have antifungal activity, and certain hydroxy fatty acids induce the production of phytoalexins, which counter pathogen invasion (Li *et al.*, 1991; Shiojiri *et al.*, 2006). This suggests that a considerable number of oxylipins could have an effect on plant physiology. In addition, OPDA has clinical implications due to the potential to provide protection against oxidative stress-related diseases (Taki-Nakano *et al.*, 2014, 2016). Given the functional diversity of the known oxylipins, these compounds may have other applications yet to be discovered.

Oxylipins are produced by fatty acid peroxidation mediated by reactive oxygen species or endogenous enzymes (Andreou *et al.*, 2009). In many plants, lipoxygenases (LOXs) have a crucial role in oxylipin biosynthesis: they catalyse the conversion of fatty acids or their esters into hydroperoxides (Nilsson *et al.*, 2012; Schuck *et al.*, 2014). LOXs are categorized as 9-LOX or 13-LOX based on the carbon position within the fatty acids that undergo peroxidation, and the products are used as substrates for further reactions (Bannenberg *et al.*, 2009). Four major downstream pathways exist: (i) the allene oxide synthase (AOS) pathway for the biosynthesis of jasmonic acid, KODA (9-hydroxy-10-oxo-12(*Z*),15(*Z*)-octadecadienoic acid), ketol, 12-oxo-phytoenoic acid, and OPDA (Yokoyama *et al.*, 2000; Itoh *et al.*, 2002; Pollmann *et al.*, 2019); (ii) the hydroperoxide lyase pathway for biosynthesis of green leaf volatiles and C12 derivatives (Matsui, 2006; Kallenbach *et al.*, 2011); (iii) the epoxy alcohol synthase and peroxygenase pathway for production of epoxy and hydroxy fatty acids (Hamberg, 1999; Hanano *et al.*, 2006); and (iv) the divinyl ether biosynthesis pathway (Fammartino *et al.*, 2007). The diverse chemical groups of the resulting products impart unique physicochemical properties and affect bioactivities.

**Fig. 1.**
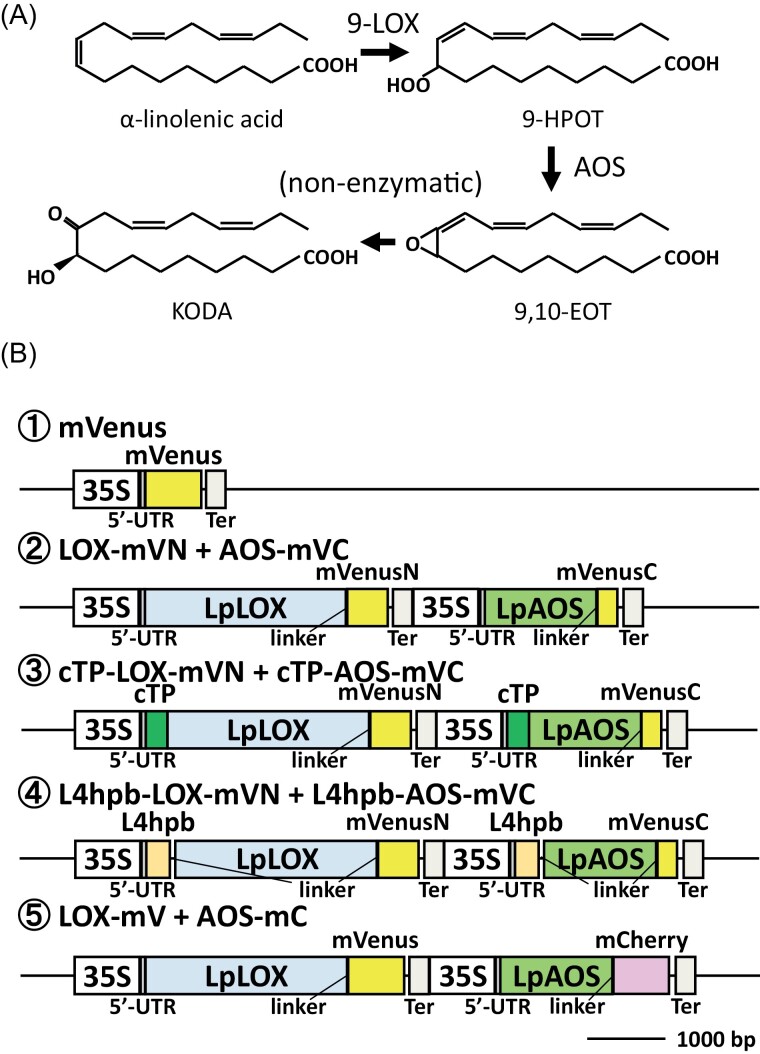
KODA biosynthesis and vector construction. (A) Biosynthesis of KODA. 9-LOX, 9-lipoxygenase; 9-HPOT, 9-hydroperoxy-10(E),12(Z),15(Z)-octadecatrienoic acid; AOS, allene oxide synthase; 9,10-EOT, 9,10-allene oxide. (B) Schematic of the constructed vectors. The boxes represent the functional areas; 35S, cauliflower mosaic virus 35S promoter; 5’-UTR, *Arabidopsis thaliana* alcohol dehydrogenase 5’-untranslated region; LpLOX, lipoxygenase of *Lemna paucicostata* strain SH; mVenusN, mVenus residues 1–173; Ter, *A. thaliana* heat-shock protein 18.2 terminator; LpAOS, allene oxide synthase of *L. paucicostata* strain SH; mVenusC, mVenus residues 156–239; cTP, *A. thaliana* RbcS residues 1–77 and the cleavage site; L4hpb, *Nannochloropsis oceanica* NIES-2145 LPAT4 residues 401–500.

KODA is an α-ketol oxylipin that is produced from α-linolenic acid via the action of 9-LOX and AOS, followed by a non-enzymatic reaction (Yokoyama *et al.*, 2000;Fig. 1A). In some plants, the intracellular concentration of KODA changes in response to environmental changes, or according to the developmental stage (Yokoyama *et al.*, 2000, 2005). The application of KODA was initially shown to promote flowering in various plants such as *Lemna paucicostata* (Yokoyama *et al.*, 2000), *Malus pamila* (Kittikorn *et al.*, 2011, 2013), *Citrus unshiu* (Nakajima *et al.*, 2016), and *Pharbitis nil* (Ono *et al.*, 2013). KODA also promotes rooting (Kawakami *et al.*, 2015), re-shooting after dormancy (Sakamoto *et al.*, 2010, 2012; Nakajima *et al.*, 2016), and it also increases wheat yield (Haque *et al.*, 2016) and enhances the resistance of certain herbaceous plants to pathogens (Endo *et al.*, 2013; Wang *et al.*, 2016, 2020). Although these findings suggest the agricultural usefulness of KODA, technical innovation is still needed to decrease the manufacturing cost, thereby ensuring a sufficient supply for practical uses of KODA in agriculture. KODA can be produced via chemical reactions (Yokokawa *et al.*, 2003) or biologically using bacteria. Indeed, KODA is commercially available (Larodan Fine Chemicals AB, Sweden). However, the manufacturing cost remains high, owing to the complex nature of the overall chemical biosynthesis scheme, and to the high cost of the starting material, α-linolenic acid, for biological production.

To address the challenges of producing KODA, we developed a system to produce KODA in plants by taking advantage of the abundance of α-linolenic acid in membrane lipids. Ectopic overexpression of two genes encoding 9-LOX and AOS in plants followed by an hour or more of incubation of leaf crude extracts was sufficient to produce a large quantity of KODA.

## Materials and methods

### Plant material

Wild-type *N. benthamiana* plants were grown at 23 °C under 23–45 µmol m^–2^ s^–1^ of continuous light. Plants were grown on a 1:1 soil mixture of PRO-MIX PGX (Premier Tech Horticulture Ltd., Canada) and vermiculite (VERMITECH, Japan) and fertilized with HYPONEX (1:2000 dilution, JAPAN). For the *Agrobacterium* transformation protocol, 4- to 6-week-old *N. benthamiana* plants were used.

Wild-type or transformants of *A. thaliana* (Columbia-0) were grown on 0.8% (w/v) agar-solidified Murashige and Skoog medium containing 1% (w/v) sucrose (Murashige and Skoog, 1962). After vernalization at 4 °C for 3 d in darkness, seedlings were grown at 23 °C under 23–45 µmol m^–2^ s^–1^ of continuous light for 10 d, and then used for experiments.

### Vector construction

The genes for *Lemna paucicostata* 9-lipoxygenase (*LpLOX*) and allene oxide synthase (*LpAOS*) were synthesized by Eurofins Genomics (Japan) according to the cDNA sequence reported in the NCBI database [HV534700 for *LpLOX*, HV534712 for *LpAOS*]. The binary vector pRI201-AN (TaKaRa, Japan) was digested with *Nde*I and *Sal*I, as recommended by the manufacturer. Subsequently, fragments containing *LpLOX*, a linker, and an N-terminal fragment of mVenus 1–173 (*mVN*) amplified by PCR were ligated into the restriction enzyme sites within the vector via the In-Fusion reaction (TaKaRa, Japan). The resulting vector was further treated with *Kpn*I and *Eco*RI to introduce PCR fragments encoding the CaMV35S promoter and Arabidopsis alcohol dehydrogenase 5’-untranslated region from pRI201-AN, *LpAOS*, a linker, a C-terminal fragment of mVenus 156–239 (*mVC*), and the heat-shock protein terminator from pRI201-AN amplified by the In-Fusion reaction. The resulting vector was named LOX-mVN/AOS-mVC (Fig. 1B). In a similar manner, the vector cTP-LOX-mVN/cTP-AOS-mVC was generated by inserting PCR fragments for a chloroplast localization sequence, N21, and the cleavage site (Shen *et al.*, 2017) at the 5’ end of *LpLOX* and *LpAOS*, and the vector L4hpb-LOX-mVN/L4hpb-AOS-mVC was generated by inserting *LPAT4hpb* (*L4hpb*) of the marine microalga *Nannochloropsis oceanica* NIES-2145, which allows the protein to localize to lipid droplets or to the endoplasmic reticulum (Nobusawa *et al.*, 2017), at each 5’ end of *LpLOX* and *LpAOS*. Similarly, the vector LOX-mV/AOS-mC was generated by introducing PCR fragments for *LpLOX*, a linker, mVenus (*mV*) at the *Nde*I site, and by introducing fragments for *LpAOS*, a linker, and mCherry from ER-rk (*mC*; Nelson *et al.*, 2007) at the *Kpn*I and *Eco*RI sites. mVenus-overexpression vector was generated to introduce mVenus into the *Nde*I and *Sal*I sites of pRI201-AN. All primers are listed in Supplementary Table S1, and all vector schemes are shown in Fig. 1B.

### *Agrobacterium* infiltration for transient gene expression in *N. benthamiana*

Vectors were introduced into *Agrobacterium tumefaciens* GV3101 by electroporation (Gene Pulser, Bio-Rad). Transformed *Agrobacteria* and viral silencing suppressor p19 (Le Mauff *et al.*, 2017) were incubated at 30 °C for 16 h in Luria-Bertani broth with antibiotics (50 µg ml^-1^ kanamycin and 25 µg ml^-1^ gentamicin). The culture was centrifuged at 2400 ×*g* for 5 min, washed once with Milli-Q water, and then resuspended in Milli-Q water. The OD_600_ was adjusted to 0.2 for p19-introduced bacteria and 0.5 for all others. Each suspension was mixed with an equal volume of the p19-containing suspension, and acetosyringone was added to a final concentration of 100 µg ml^-1^ before infiltration. The abaxial surface of *N. benthamiana* leaves was infiltrated using a plastic syringe.

### Transformation of *A. thaliana* with *Agrobacterium*

Transgenic lines of *A. thaliana* (Columbia ecotype) expressing mVenus or cTP-LOX-mVN/cTP-AOS-mVC were generated using the floral dip method (Clough and Bent, 1998). The transformants were selected by tolerance to 25 µg ml^-1^ kanamycin. Expression of the transgenes was observed by mVC fluorescence (Supplementary Fig. S1).

### Sample preparation for quantification of KODA

To purify KODA from *N. benthamiana* and *A. thaliana*, leaves and shoot tissues were frozen in liquid nitrogen and then crushed in MilliQ water (1 ml per 6.7 mg fresh weight of *N. benthamiana*, and 1 ml per 4 mg fresh weight of *A. thaliana*) to obtain crude extracts. The leaf crude extracts were incubated for 1 h at 23 °C or on a heating block at 15 °C, 25 °C, 35 °C, or 45 °C. The cell debris was separated by centrifugation at 16 400×*g* for 2 min, and each resultant supernatant was supplemented with formic acid in methanol to achieve a final formic acid concentration of 1% (v/v). Each mixture was passed sequentially through a MonoSpin^®^ Phospholipid (GL Sciences Inc., Japan) and a Millex-GV Durapore (0.22 µm pore size, 4 mm diameter, polyvinylidene difluoride, Merck KGaA, Germany) column. The flow-through solutions were used for KODA measurements.

### Quantification of KODA by liquid chromatography coupled with tandem mass spectrometry

Chromatographic separation was carried out with an LC-2040C 3D system (Shimadzu, Japan) using a Kinetex Polar C18 column (2.1 × 100 mm) with a 2.6 μm particle size (Phenomenex, USA). Mass spectrometric detection was performed with an LCMS-8050 tandem quadrupole mass spectrometer (Shimadzu, Japan). The flow rate was 0.4 ml min^−1^. The following linear-gradient program was used with solvents A (0.1% formic acid) and B (acetonitrile): 0 min, 30% B; 5 min, 35% B; 10 min, 50% B; 20 min, 75% B; 20.1 min, 95% B; 25 min, 95% B; 26 min, 30% B; and 31 min, 30% B. The effluent from the analytical column was routed directly into the electrospray ionization (ESI) source of the mass spectrometer. The nebulizing gas flow was set to 3 l min^–1^, heating gas flow to 10 l min^–1^, and drying gas flow to 10 l min^–1^. The collision-induced dissociation gas pressure was set to the tuning file created by the manufacturer. The interface temperature was set to 300 °C, Desolvent Line (DL) temperature to 250 °C, and block heater temperature to 400 °C. KODA was measured by setting the ion source to –3 kV (ESI negative), the column elution time to 10.1–14 min, the m/z transition to 309.2 → 200.2, and the Q1 and Q3 resolution to ‘Low’ (preset value: 4800). In addition, the Q3 scan was monitored by setting the ion source to –3 kV (ESI negative), the time to 10–14 min, and m/z to 150–1000, and MRM was used for instrumental stability by setting the ion source to 4 kV (ESI positive), the time to 16–17 min, and the m/z transition to 900 → 800.

All system controls and data analyses were processed with LabSolution software (Shimadzu, Japan).

### Imaging of transformants by laser-scanning confocal microscopy

Images of the leaves of tobacco plants 3 d after *Agrobacterium* infiltration were acquired by laser-scanning confocal microscopy LSM780 (Carl Zeiss, Germany) and a water lens W Plan-Apochromat 40×/1.0 differential interference contrast microscope (M27, Carl Zeiss). mVenus fluorescence was excited at 514 nm (laser intensity: 1.5%, emission: 519–560 nm, pinhole: 47 µm), mCherry fluorescence was excited at 561 nm (laser intensity: 1.5%, emission: 578–630 nm, pinhole: 51 µm), and chlorophyll autofluorescence was excited at 633 nm (laser intensity: 0.9%, emission: 647–688 nm, pinhole: 48 µm). Images were processed with ZEN imaging software (Carl Zeiss, Germany), and all images were created using the same display parameters.

## Results

**Fig. 2.**
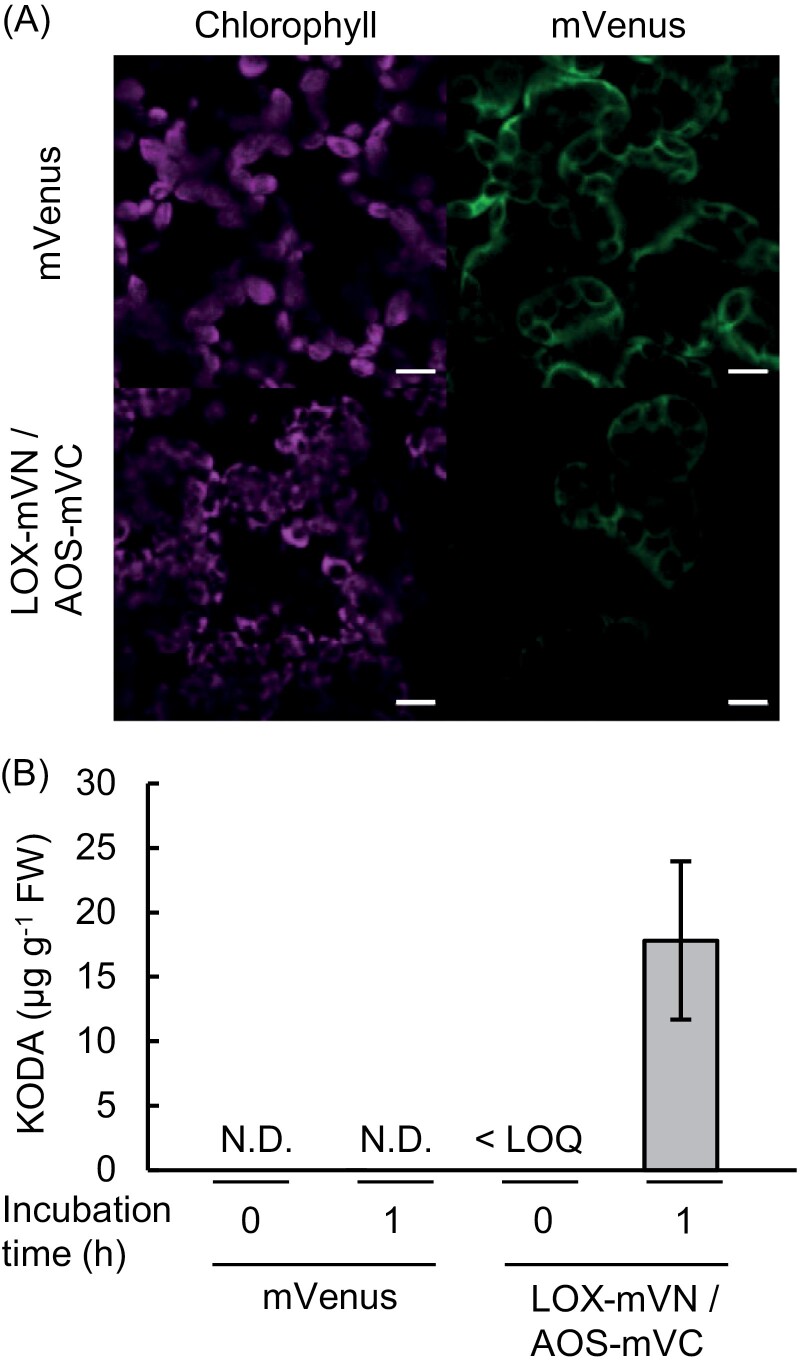
Transient expression of mVenus or LOX-mVN/AOS-mVC in leaves of *Nicotiana benthamiana*. (A) Sub-cellular localization of mVenus fluorescence in leaves expressing mVenus (upper panel) or LOX-mVN/AOS-mVC (lower panel) visualized by laser-scanning confocal microscopy. Chlorophyll, chlorophyll autofluorescence. Scale bars =10 µm. (B) KODA content in *N. benthamiana* leaves transiently expressing mVenus or LOX-mVN/AOS-mVC. KODA in leaf crude extracts was quantified after a 1 h incubation (grey bar) or no incubation (white bar). ND, not detected; LOQ, limit of quantification; FW, fresh weight. Values represent the mean ±SD (*n=*3).

To develop a system for *in vivo* production of large quantities of KODA, we used 9-LOX (HV534700) and AOS (HV534712) of *L. paucicostata* strain SH which have high activities to produce KODA (Yokoyama and Beppu, 2011). It has been reported that LOX, AOS, and allene oxide cyclase form a complex that is utilized in the jasmonic acid biosynthetic pathway of Arabidopsis (Pollmann *et al.*, 2019). Therefore, we modified *L. paucicostata* LOX (LpLOX) and LpAOS to enhance their interaction, and thereby promote heterodimer formation (Fig. 1B). The system utilized partially overlapping mVenus coding sequences. The encoded fragment pair comprised amino-acid residues 1–173 and 155–239 of the fluorescent protein mVenus, as the fragments interact strongly (Gookin and Assmann, 2014). This suggested that the physical association between the 1–173 fragment and 155–239 fragment of mVenus would result in Venus fluorescence. Transient expression of LOX harbouring amino acid residues 1–173 of mVenus (LOX-mVN) and AOS harbouring amino acid residues 155–239 of mVenus (AOS-mVC) in leaves of *Nicotiana benthamiana* resulted in Venus fluorescence in the cytoplasm (Fig. 2A), indicating that the two proteins associated in the cytoplasm. However, KODA content in the leaf crude extract was below the quantification limit (Fig. 2B; 0 h). Given that 9-LOX has substrate preference for free fatty acids, especially free α-linolenic acid (Liavonchanka and Feussner, 2006) that is usually present in small quantities in plant leaves, the low abundance of α-linolenic acid was considered one possibility for the observed low amount of KODA in our *in vivo* system. Although the cellular concentration of free fatty acids is low under normal growth conditions, it markedly increases in plant leaves after wounding, possibly owing to the enhanced breakdown of membrane glycerolipids (Conconi *et al.*, 1996). Thus, we investigated KODA accumulation in leaf crude extracts after a 1 h incubation at 23 °C, which promoted the enzymatic breakdown of those membrane glycerolipids containing α-linolenic acid (Fig. 2B; 1 h, grey bars). This 1 h incubation resulted in accumulation of KODA, i.e. >1.8 µg g^-1^ fresh weight, in leaf crude extracts of *N. benthamiana* expressing LpLOX and LpAOS, whereas little KODA accumulated in crude extracts of leaves expressing only mVenus or crude extracts that were not incubated (Figs 2B, 4; 1 h). These results suggested that expression of LpLOX and LpAOS, as well as subsequent incubation of the leaf crude extract, was essential for producing substantial amounts of KODA in plant leaves.

**Fig. 3.**
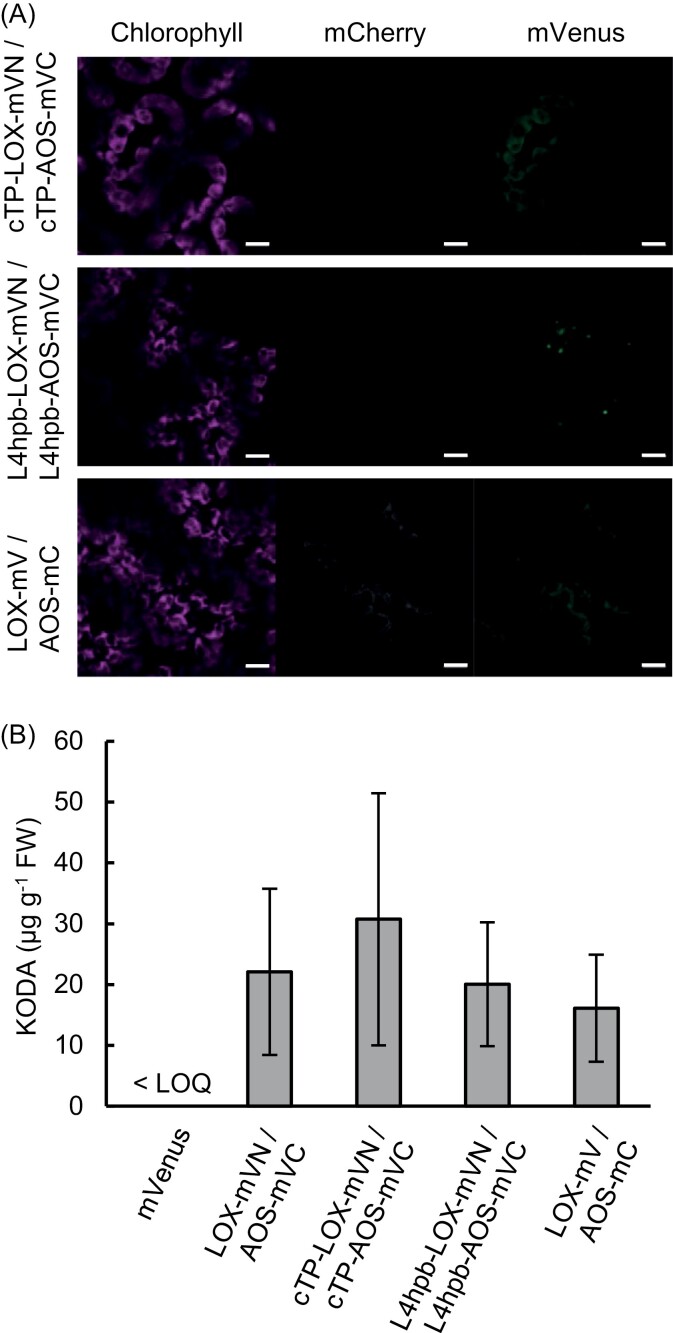
Transient expression of LOX and AOS in leaves of *Nicotiana benthamiana*. (A) Sub-cellular localization of mVenus or mCherry fluorescence in leaves expressing cTP-LOX-mVN/cTP-AOS-mVC, L4hpb-LOX-mVN/L4hpb-AOS-mVC, or LOX-mV/AOS-mC. Fluorescence was observed by laser scanning confocal microscopy. Chlorophyll, chlorophyll autofluorescence. Scale bars =10 µm. (B) KODA content in *N. benthamiana* leaves transiently expressing mVenus, LOX-mVN/AOS-mVC, cTP-LOX-mVN/cTP-AOS-mVC, L4hpb-LOX-mVN/L4hpb-AOS-mVC, or LOX-mV/AOS-mC. KODA content in leaf extracts was quantified after a 1 h incubation. LOQ: limit of quantification; FW, fresh weight. Values represent the mean ±SD (*n*=3).

Chloroplast membranes store an abundance of α-linolenic acid. Indeed, α-linolenic acid that is esterified to glycerolipids in chloroplast membranes is a substrate for jasmonic acid biosynthesis (Wang *et al.*, 2019). To determine whether the sub-cellular localization of 9-LOX and AOS affects the efficiency of KODA production, we analysed KODA content in leaf crude extracts of *N. benthamiana* transiently expressing plastid-localized 9-LOX and AOS (cTP-LOX-mVN/cTP-AOS-mVC) and, for comparison, endoplasmic reticulum– or lipid droplet–localized 9-LOX and AOS (L4hpb-LOX-mVN/L4hpb-AOS-mVC; Figs 1B, 3). The localization of the 9-LOX/AOS heterodimer was documented based on Venus fluorescence; the results revealed that chloroplast-targeted 9-LOX and AOS localized to chloroplasts, and that lipid droplet–targeted 9-LOX and AOS localized to the endoplasmic reticulum/lipid droplets (Fig. 3A). KODA content in leaf crude extracts after a 1 h incubation did not differ markedly among these transformants, and this was also the case for the cytoplasmic localization of 9-LOX and AOS (LOX-mVN/AOS-mVC; Fig. 3B), suggesting that localization of the 9-LOX/AOS heterodimer did not correlate with KODA content. Moreover, we also analysed the cytoplasmic localization of 9-LOX and AOS that were not tethered to mVenus fragments (Fig. 3B; LOX-mV/AOS-mC), which yielded results similar to those obtained with the mVenus constructs. This suggested that the attachment of mVenus fragments to the 9-LOX/AOS heterodimer was not necessary for KODA production mediated by the transient expression system in *N. benthamiana*.

**Fig. 4.**
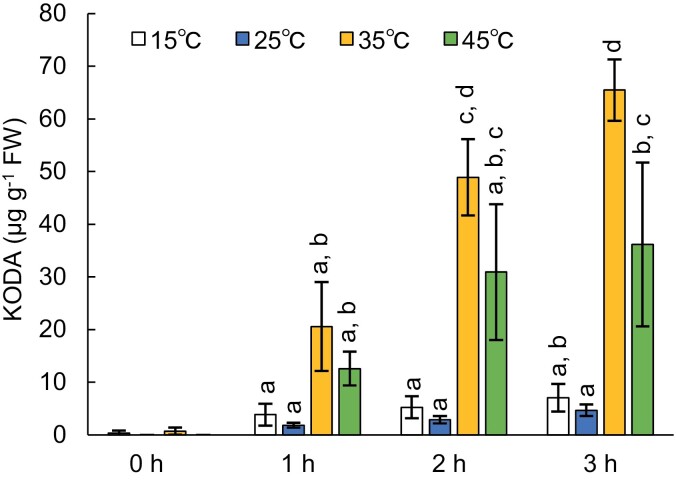
Effect of incubation time and temperature on KODA content in leaves of *Nicotiana benthamiana*. LOX-mVN and AOS-mVC were transiently expressed in leaves of *N. benthamiana* and KODA content measured after 0, 1, 2, or 3 h incubation at 15, 25, 35, or 45 °C. Values represent the mean ±SD (*n*=3). Different lowercase letters indicate that KODA content differed significantly (*P*<0.05, Tukey-Kramer test for time points 1, 2, and 3 h for each sample). FW, fresh weight.

We next optimized both the temperature and duration of the incubation period with respect to KODA production, in leaf crude extracts from *N. benthamiana* transiently transformed with LOX-mVN and AOS-mVC (Fig. 4). KODA content was greater when crude extracts were incubated at 35 °C or 45 °C for 2 h or 3 h, compared with incubation at 15 °C or 25 °C (Fig. 4). KODA might be rapidly degraded *in vivo* owing to its propensity to undergo chemical reduction, hinting that longer incubation periods might reduce KODA content. However, KODA content in the leaf crude extract increased with incubation time up until 3 h at all temperatures, suggesting that the rate of KODA biosynthesis was much greater than the rate of degradation in the leaf crude extract of *N. benthamiana*.

**Fig. 5.**
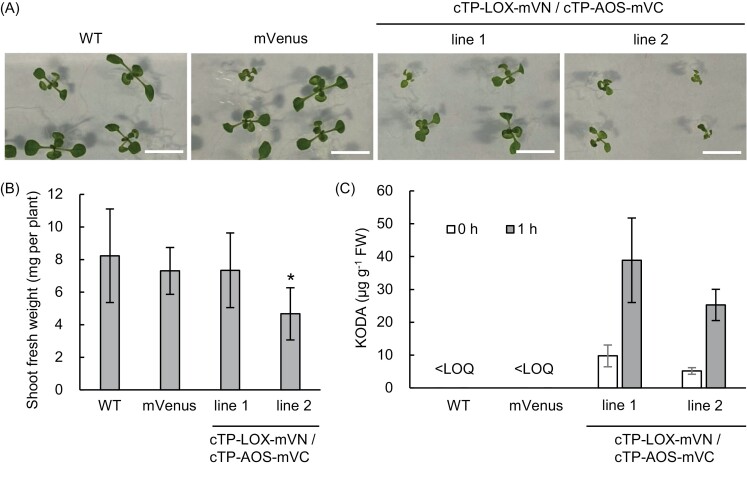
Growth phenotype and KODA content in Arabidopsis transformants expressing cTP-LOX-mVN and cTP-AOS-mVC. (A) Growth of Arabidopsis wild-type (WT) and transformants expressing mVenus (mVenus), or transformants expressing cTP-LOX-mVN and cTP-AOS-mVC (cTP-LOX-mVN/cTP-AOS-mVC line 1 and line 2, plastid-targeted) grown for 10 d on Murashige and Skoog medium under continuous light. Scale bars =1 cm. (B) Shoot fresh weight (FW) of 10-day-old wild-type (WT), Arabidopsis transformants expressing mVenus (mVenus), or transformants expressing cTP-LOX-mVN and cTP-AOS-mVC (cTP-LOX-mVN/cTP-AOS-mVC line 1 and line 2, plastid-targeted). Data are means ±SD, *n=*6, biological repeats; Steel’s test, ∗*P*<0.05. (C) KODA content in shoots. Data are means ±SD, *n=*3. LOQ, limit of quantification.

Establishing a KODA production system in plants requires stable transformants that produce KODA, without any negative effects on plant growth. Thus, we introduced chloroplast-localized 9-LOX and AOS into *Arabidopsis thaliana*, and obtained two lines of transformants (Figs 1B, 5; cTP-LOX-mVN/cTP-AOS-mVC). Compared with wild-type plants and mVenus-expressing transformants, both transformant lines appeared smaller at 10 d (Fig. 5A). Indeed, the shoot fresh weight of the wild type, mVenus-expressing, and line 1 transformants were comparable, although that of line 2 was markedly lower by comparison (Fig. 5B). KODA content was measured using leaves from 10-day-old plants, which demonstrated that the stable transgenic Arabidopsis plants were also able to produce KODA in leaves (Fig. 5C). Incubation of the Arabidopsis leaf crude extracts was also important to increase KODA production (Fig. 5C). Notably, Arabidopsis transformants expressing cytoplasm-localized 9-LOX and AOS could not be obtained after screening for kanamycin tolerance (Fig. 1B; LOX-mVN/AOS-mVC). Given that the cytoplasm-localized enzymes were active when transiently expressed in *N. benthamiana*, cytoplasmic localization of 9-LOX and AOS might negatively affect plant growth and perhaps seed germination and/or seedling establishment as well. These results suggested that ectopically expressed 9-LOX and AOS should not be localized in cytoplasm but must be targeted to chloroplasts, and that leaf crude extracts must be incubated at room temperature (23 °C , or preferably 35 °C) for up to 3 h, to achieve maximum KODA production. Thus, this system may be applicable to other plant species, considering that it was successful in two distinct plant species, namely *N. benthamiana* and *A. thaliana*.

## Discussion

We introduced two enzymes of *L. paucicostata* strain SH for KODA biosynthesis into *N. benthamiana* and *A. thaliana.* The two enzymes, namely 9-LOX and AOS, harbouring the N- and C-terminal fragments of mVenus, respectively, at the C-terminus of each protein, were expected to efficiently form a heterodimer, according to previous findings (Gookin and Assmann, 2014). Indeed, the expressed proteins formed a heterodimer in the cells of transiently transformed tobacco plants, as documented by mVenus fluorescence (Figs 2A, 3A). This system for heterodimer formation was applicable to proteins that localized in the cytoplasm, chloroplasts, and the endoplasmic reticulum or lipid droplets, suggesting that the system could be applied to any proteins, regardless of their sub-cellular localization (Fig. 3A). Thus, the system constitutes an effective means of promoting the formation of protein complexes in living cells, because only a short mVenus sequence must be added to each protein, and complex formation and sub-cellular localization can be easily verified by Venus fluorescence.

Our system could produce KODA in leaf crude extracts of *N. benthamiana* and *A. thaliana*, although there appeared to be no correlation between KODA content and formation of the 9-LOX/AOS heterodimer. Thus, heterodimerization of 9-LOX and AOS might not be necessary for KODA biosynthesis if these proteins were to be highly expressed, and the substrate was abundant. Future work must address the correlation between heterodimerization of 9-LOX and AOS, and KODA production.

Each of the *N. benthamiana* and *A. thaliana* transformants accumulated substantial amounts of KODA only when the leaf crude extracts were incubated for an hour or more (Figs 1B, 5B). Moreover, KODA content increased with incubation time of up to 3 h (Fig. 4). The fact that maximal KODA production was achieved with incubation at 35 °C suggests that several enzymatic reactions contributed to KODA biosynthesis during the incubation period. Notably, the concentration of the substrate, namely free α-linolenic acid, may have increased via degradation of membrane and storage glycerolipids in leaf crude extracts during the incubation, and this increased abundance of α-linolenic acid may have enhanced KODA production. However, the fact that only small amounts of KODA were produced in crude extracts without incubation suggests that KODA concentration in leaves is mainly regulated by β-oxidization or other lipid degradation processes. Interference with KODA degradation during the incubation period might also have contributed to KODA accumulation. In any case, KODA accumulation in living cells might require some type of stress that induces glycerolipid degradation (i.e. to supply α-linolenic acid) and/or curbs KODA degradation. This also explains why KODA contributes to recovery of plants from stress (Haque *et al.*, 2016).

Intriguingly, we could not generate Arabidopsis transformants that expressed cytoplasm-localized 9-LOX and AOS, although transformants that expressed chloroplast-localized 9-LOX and AOS were generated without any problem. In chloroplasts, free fatty acids produced via glycerolipid breakdown immediately to interact with phytol, and are converted to fatty-acid phytyl esters by phytyl ester synthase, perhaps to avoid the accumulation of the toxic compounds in chloroplasts (Lippold *et al.*, 2012). However, the mechanism for cytoplasmic conversion of free fatty acids to relatively stable forms, such as fatty acid phytyl esters, has been not determined. Moreover, during germination, triacylglycerols stored as carbon and energy sources in seeds are degraded, and the subsequent biosynthesis of membrane glycerolipids accelerates to establish intracellular membranes—especially in photosynthetic tissues. Indeed, during the process of lipid turnover in germinating seeds or seedlings, the concentration of free linolenic acid might increase sufficiently so as to provide abundant substrate for KODA biosynthesis. Vellosillo *et al.* (2007) reported that several oxylipins produced by 9-LOX perturbs root development during germination. In maize, 9-LOX-derived linolenate oxidation products, including 10-oxo-11-phytoenoic acid, display direct phytoalexin activity against biotic agents, mediate defence gene expression, and can promote cytotoxicity resulting in cell death (Christensen *et al.*, 2015, 2016). Thus, with regard to KODA production in stable transgenic plants, it is important to consider the sub-cellular localization of ectopically expressed LOX and AOS.

Our results constitute a new method for producing compounds that require serial reactions of enzymes, for which the initial substrates are fatty acids that must be cleaved from glycerolipids of plants. This method could be used for mass production of other oxylipins, which would facilitate research towards the goal of including oxylipins in fertilizers and/or pesticides. The amount of KODA produced in the transgenic plants might be still low for further use, because KODA needs to be purified from the leaf crude extracts, and a method has not yet been established. The transgenic plants would however, be useful to study the effect of *in planta-*produced KODA on plant physiology, including the synthesis of the other stress hormones.

## Supplementary data

The following supplementary data are available at *JXB* online.

Table S1. Primers for vector construction.

Fig. S1. Sub-cellular localization of mVENUS fluorescence in Arabidopsis wild type and transformants.

erab557_suppl_Supplementary_Table_S1

## Data Availability

The data supporting the findings of this study are available from the corresponding author, Mie Shimojima, upon request.

## Abbreviations

AOS: allene oxide synthase
KODA: 9-hydroxy-10-oxo-12(Z),15(Z)-octadecadienoic acid
LOX: lipoxygenase
OPDA: 12-oxo-phytodienoic acid
