# Supplementary material for: Developing a platform for production of the oxylipin KODA in plants
Source: J Exp Bot. 2021 Dec 27;73(9):3044–52. doi: 10.1093/jxb/erab557 (PMC9113317; doi:10.1093/jxb/erab557)
Supplement: erab557_suppl_Supplementary_Table_S1 [file erab557_suppl_supplementary_table_s1.pdf]

Table. S1 Primers for vector construction.

Vector name	Description	Sequence
LOX-mVN / AOS-mVC	pRI201MCS1-LpLOX_F	CACTGTTGATACATAATGGCCGGTTTTCTCCAA
	LpLOX-linker-mVenusN_R	TCTTCACCTTTAGACACCATACTCCCACCTCCACCAATGGAGATGCTGTTGGGGAT
	mVenus_F	ATGGTGTCTAAAGGTGAAGA
	mVenusN-pRI201MCS1_R	ATTCAGAATTGTCGATTACTCTATGTTATGTCTGATCTTGAA
	pRI201MCS2-CaMV35S_F	AAAGATAAGGAATTGAGATTAGCCTTTTCAATTTCAGAA
	ADH5'UTR_R	TATGTATCAACAGTGAAGAACTTG
	ADH5'UTR-LpAOS_F	TCTTCACTGTTGATACATAATGTCTGTCTCGCAATCAG
	LpAOS-linker-mVenusC_R	ATCCCGTTCTTCTGTTTATCACTTCCACCTCCACCTCGGGTCGTCGCCTTCG
	mVenusC_F	GATAAACAGAAGAACGGGA
	mVenusC-HSPterminator_R	GAATTGTCGACCCGGTAGTATTACTTGTAAAGCTCATCCATG
	HSPterminator_F	TACTACCGGGTCGACAAT
	HSPterminator-pRI201MCS2_R	CCATGATTACGAATTCTTATCTTTAATCATATTCCATAGTCCA
cTP-LOX-mVN / cTP-AOS-mVC	pRI201MCS1-AtRbcScTP_F	CACTGTTGATACATAATGGCTTCCTCTATGCTC
	AtRbcScTP-N21-cleavagesite_R	ACATCGTATTCTCCCTCCGTTGGACACATTTCCGAAGCTGCTATTTCCGGTAAGGTCA GGAAGGTAAG
(not listed above)	AtRbcScTP-LpLOX_F	ACGGAGGGAGAATACGATGTATGGCCGGTTTTCTCCAA
L4hpb-LOX-mVN / L4hpb-LOX-mVC	pRI201MCS1-LPAT_F	CACTGTTGATACATAATGCAGACGCAGAGTTTTAGGGGGGAGAAGGA
	LPAT-linker_R	CCCTTGGGTCGATCCTCCTCCTGAACCACCACTACCACCCGATCCTCCG CCGCCGTCAGCGGAATTACCGGTCCGTTGCTGGTGCTGGTGCCGGTGT
(not listed above)	linker-LpLOX_F	GAGGAGGATCGACCCAAGGGATGGCCGGTTTTCTCCAA
	linker-LpAOS_F	GAGGAGGATCGACCCAAGGGATGTCTGTCTCGCAATCAG
LOX-mV / AOS-mC (not listed above)	mVenus-pRI201MCS1_R	TAATCGACGGGCATATTACTTGTAAAGCTCATCCATGC
	LpAOS-mCherry_R	TCCTCGCCCTTGCTCACCATACTTCCACCTCCACCTCGGGTCGTCGCCTTCG
	mCherry_F	ATGGTGAGCAAGGGCGAGGA
	mCherry-HSPterminator_R	GAATTGTCGACCCGGTAGTATTACTTGTACAGCTCGTCCATGC

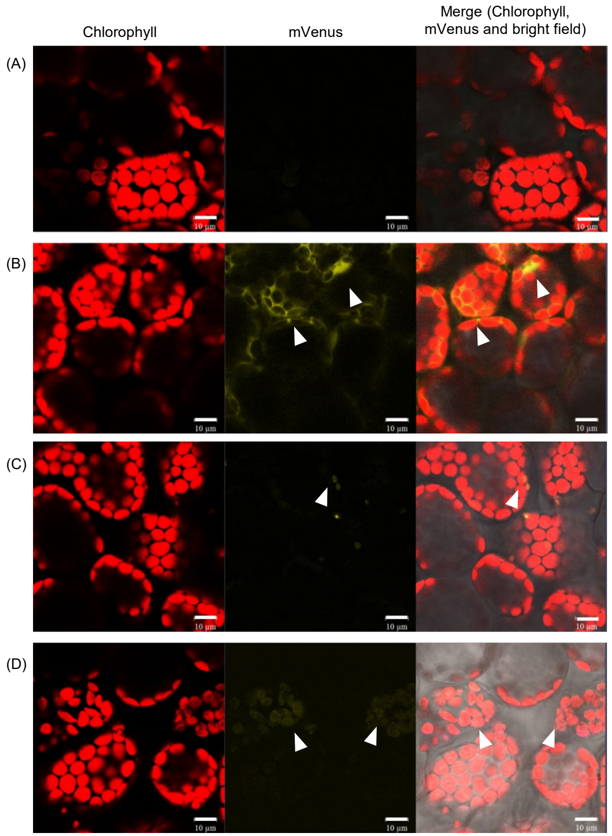

Supplementary Figure S1. Sub-cellular localization of mVenus fluorescence in leaves of Arabidopsis wild-type (A), transformants expressing mVenus (B) and transformants expressing cTP-LOX-mVN and cTP-AOS-mVC (cTP-LOX-mVN/cTP-AOS-mVC line 1 (C) and line 2 (D), plastid-targeted) grown for 10 days on Murashige and Skoog medium under continuous light. Fluorescence was observed by laser scanning confocal microscopy. Chlorophyll, chlorophyll autofluorescence. White arrow heads indicate mVenus fluorescence. Scale bars =10 µm.
